# Who influences nutrition policy space using international trade and investment agreements? A global stakeholder analysis

**DOI:** 10.1186/s12992-021-00764-7

**Published:** 2021-10-02

**Authors:** Kelly Garton, Boyd Swinburn, Anne Marie Thow

**Affiliations:** 1 School of Population Health, The University of Auckland, Auckland, New Zealand; 2 Menzies Centre for Health Policy, The University of Sydney, Sydney, NSW Australia

**Keywords:** International trade and investment, Policy space, Nutrition policy, Food systems governance, Stakeholder analysis, Advocacy

## Abstract

**Background:**

Regulation of food environments is needed to address the global challenge of poor nutrition, yet policy inertia has been a problem. A common argument against regulation is potential conflict with binding commitments under international trade and investment agreements (TIAs). This study aimed to identify which actors and institutions, in different contexts, influence how TIAs are used to constrain policy space for improving food environments, and to describe their core beliefs, interests, resources and strategies, with the objective of informing strategic global action to preserve nutrition policy space.

**Methods:**

We conducted a global stakeholder analysis applying the Advocacy Coalition Framework, based on existing academic literature and key informant interviews with international experts in trade and investment law and public health nutrition policy.

**Results:**

We identified 12 types of actors who influence policy space in the food environment policy subsystem, relevant to TIAs. These actors hold various beliefs regarding the economic policy paradigm, the nature of obesity and dietary diseases as health problems, the role of government, and the role of industry in solving the health problem. We identified two primary competing coalitions: 1) a ‘public health nutrition’ coalition, which is overall supportive of and actively working to enact comprehensive food environment regulation; and 2) an ‘industry and economic growth’ focussed coalition, which places a higher priority on deregulation and is overall not supportive of comprehensive food environment regulation. The industry and economic growth coalition appears to be dominant, based on its relative power, resources and coordination. However, the public health nutrition coalition maintains influence through individual activism, collective lobbying and government pressure (e.g. by civil society), and expert knowledge generation.

**Conclusions:**

Our analysis suggests that industry and economic growth-focussed coalitions are highly capable of leveraging networks, institutional structures and ideologies to their advantage, and are a formidable source of opposition acting to constrain nutrition policy space globally, including through TIAs. Opportunities for global public health nutrition coalitions to strengthen their influence in the support of nutrition policy space include strategic evidence generation and coalition-building through broader engagement and capacity-building.

**Supplementary Information:**

The online version contains supplementary material available at 10.1186/s12992-021-00764-7.

## Background

Over the past 25 years since the inception of the World Trade Organization (WTO), the proliferation of international trade and investment in food and beverage markets has shaped global food environments inexorably [1–5]. A shift toward greater access to, availability and consumption of processed and ultra-processed foods has been accompanied by a rise in malnutrition in all its forms, including obesity, and dietary non-communicable diseases (NCDs) [6–9]. In order to curb the global burden of malnutrition and NCDs, the World Health Organization recommends government action to improve the healthiness of food environments, including through fiscal policies, front-of-pack nutrition labelling, restricting marketing to children, and limiting the level of sodium, trans fats and other nutrients in the food supply [10–12].

Scholarship in the past decade has raised concerns that trans-national companies (TNCs) and other opposed parties may use international trade and investment agreements (TIAs) as a means to contest the legitimacy of such regulations and restrict governments’ ‘policy space’ for food environment (nutrition) interventions [13–24]. Policy space refers to “the freedom, scope, and mechanisms that governments have to choose, design, and implement public policies to fulfil their aims.” [13] Several recent studies have highlighted the key role of power, and its asymmetrical distribution between stakeholders, in enabling such restriction to occur [25–29]. As agents who use and interpret TIAs in different ways, a variety of actors and institutions play a role in potentially constricting policy space—specifically by influencing whether and how TIA mechanisms of nutrition policy space constraint are activated, or not [30]. This may be, for example, through technical challenges on the basis of specific trade agreements, such as claims that regulations are discriminatory against other ‘like’ products, are more trade-restrictive than necessary as technical barriers to trade, or violate the rights given to investors to protect their investments (e.g. amounting to ‘indirect expropriation’ or lack of ‘fair and equitable treatment’) [31–33]. There may also be appeals to associated neoliberal values in trade and investment forums, [34] in what Smith (2020) calls the ‘political determinants of health.’ [35] A more detailed understanding of the roles, interests, relationships and resources of the various stakeholders is necessary to inform strategic action to preserve nutrition policy space.

This stakeholder analysis aimed to examine how actors and institutions, in different contexts, influence TIA-related mechanisms of policy space constraint for improving food environments. The analysis was guided by three main research questions:

1. Who are the actors and institutions relevant to the (global) food environment policy subsystem?
2. How do they influence policy space through TIA mechanisms?
3. What are the important networks and power dynamics?

## Methods

### Data sources

The data was obtained from 26 literature sources and 22 interviews collected by the lead author as part of their PhD thesis. Documentary sources in the form of academic literature, institutional reports, trade and investment dispute documents, and WTO Committee meeting minutes were identified through a systematic search of 5 academic databases, 13 institutional websites, and 4 dispute databases, and detail of the search strategy is reported in a previous publication [30]. Interviews were held with global experts in international trade and investment law, public health researchers, government bureaucrats working in public health and trade policy, and representatives from inter-governmental organisations concerned with global health, nutrition, and trade and investment (Table 1). These were selected purposively based on the authors’ knowledge of the (relatively small) pool of global expertise in this field, and augmented by snowballing. Semi-structured interviews focused on three key nutrition policy areas: front-of-pack nutrition labelling, restricting marketing to children, and nutrient content limits. Interview scripts were structured as ‘vignettes’ to explore potential policy space outcomes based on a series of changing regulatory scenarios, an example of which is published elsewhere [36]. All responses pertaining to actors, stakeholders, relationships, power dynamics, and agency in TIA mechanisms of influence on nutrition policy space were collected and included in this stakeholder analysis. We anonymised participant responses with the labels P1 – P22.

**Table 1:** Interview participant characteristics

Participant characteristics (*N* = 22)			
Policy area interviewed	Geographic region	Sector	Discipline
Labelling (n=9) Marketing (n=9) Nutrient composition (n=4)	Australasia (n=9) Latin America & the Caribbean (n=7) Europe & UK (n=3) North America (n=2) Sub-Saharan Africa (n=1)	Non-government organisation (n=19) Academic (n=16) Public sector (n=2) Private sector (n=1) Inter-government organisation (n=1)	Trade law (n=12) Investment law (n=7) Public health nutrition (n=8)

*Note*: while we have shown the regional distribution of participants, most had global expertise and perspectives. Characteristics of policy area interviewed and region are discrete, but participants often belonged to more than one sector and were working under more than one discipline

### Theory

Our approach to data organisation was guided by Varvasovsky & Brugha’s (2000) practical how-to manual for stakeholder analysis [37]. The analysis was underpinned by Sabatier’s Advocacy Coalition Framework (ACF), which conceptualises coalitions of actors (stakeholders), brought together by shared beliefs, as being influential in shaping policy outcomes for a given policy area [39]. We also drew upon theories of power from Lukes (1974) to further describe the sources and types of power wielded by actors within the policy system [40].

According to the ACF, an ‘advocacy coalition’ is a network of stakeholders from various public and private organisations who are actively concerned with the maintenance and evolution of policy in a particular domain [41]. We defined ‘stakeholder’ as any actor or institution that can influence, and may be impacted by, public health nutrition / food environment regulation policy space with respect to TIAs [37]. Core policy beliefs we defined as:

the priority of different policy-related values, whose welfare counts, the relative authority of governments and markets, the proper roles of the general public, elected officials, civil servants, experts, and the relative seriousness and causes of policy problems in the subsystem as a whole.( [32] p195)

**Fig. 1:**
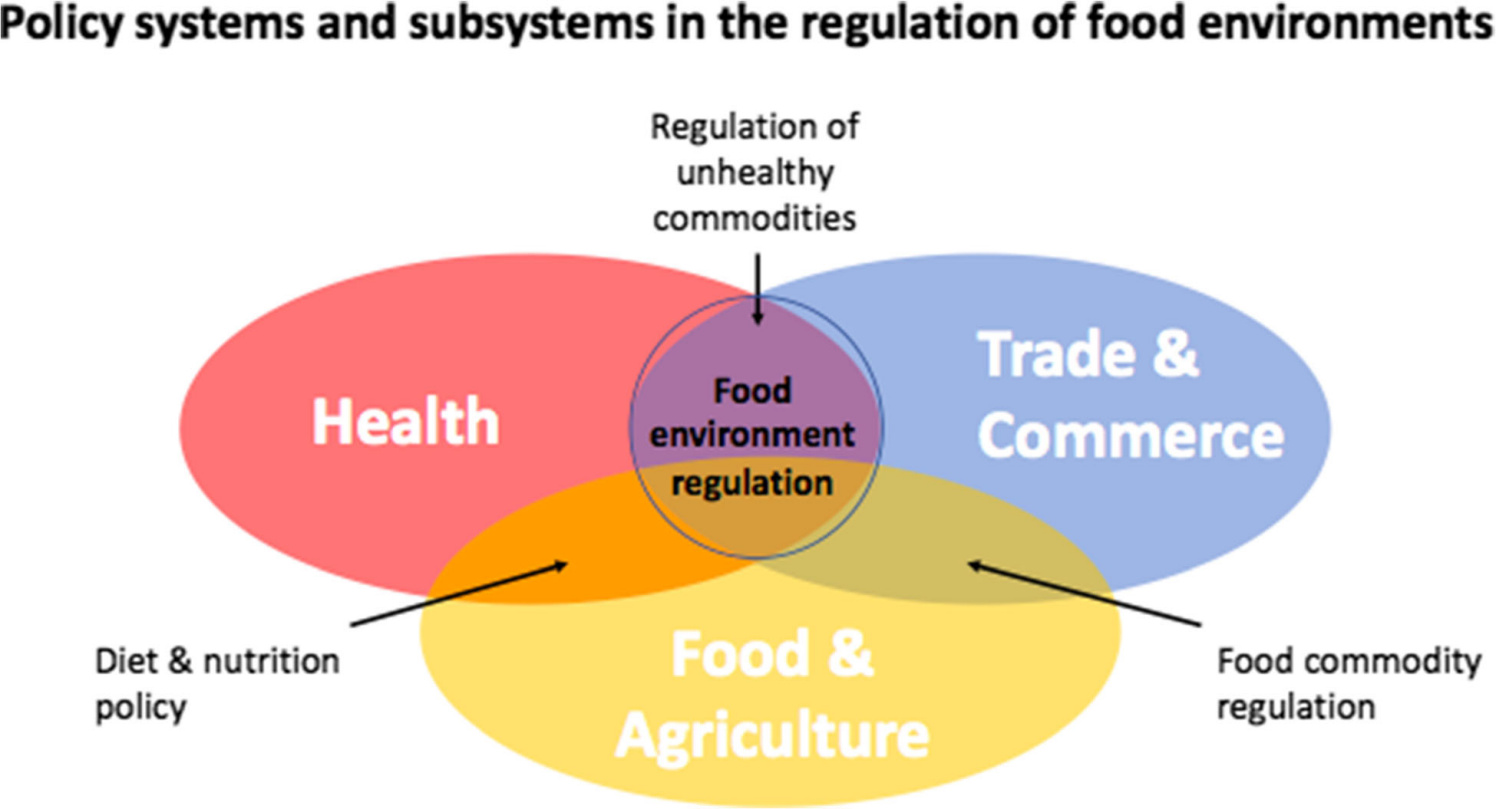
Intersection between policy systems and subsystems involved in the regulation of food environments for NCD prevention Note: This particular area of intersection (circled) is selected (not just the combination of all three) because the food and beverage products of interest in this study are manufactured processed and ultra-processed foods, many of which fall outside the sector of food and agriculture in the traditional sense and thus relate (only) to the intersection between health and trade/commerce policy

These stakeholders are embedded in the various relevant ‘policy systems’, within both the public and private sectors. Regulation of food environments for NCD prevention involves the intersection of at least three policy systems: health, trade/commerce, and food/agriculture (Fig. 1). At their intersection, food environment regulation forms a policy ‘subsystem,’ i.e. the domain and set of policies, and associated processes and institutions, that these stakeholders are seeking to influence. For this study, the policy subsystem frame-of-reference was defined as the food environment policy subsystem (and associated ‘nutrition policy space’). We examined how various stakeholders factor into policy space at the intersection between nutrition policy, specifically the types of food environment regulation mentioned above, and international trade and investment policy.

A ‘policy subsystem’ is therefore heterogeneous, containing more than one advocacy coalition, of which there are dominant and non-dominant advocacy coalitions [41]. The dominant coalition has its beliefs largely reflected in the existing policy, and thus generally seek to maintain the status quo. The non-dominant coalition(s) seek to block or change the policy subsystem. There is therefore always a dynamic tension between two to three advocacy coalitions, sometimes resulting in shocks or ‘perturbations’ in the policy subsystem [42].

In terms of how to structure the data for analysis, Varvasovsky & Brugha’s practical approach to stakeholder analysis involves collecting and organising data for each stakeholder-type according to: involvement in the issue (in this case, food environment regulation and policy space with respect to TIAs), interest in the issue, influence/power in the issue, position with respect to the issue, and impact of the issue on the stakeholder.

To further tease out sources and types of power and influence present in the policy subsystem, we drew upon Lukes’ three dimensions of power including visible/direct influencing of decision making, institutional bias (i.e. the ‘rules of the game’), and implicit/ideological underlying core beliefs that shape how actors consider policy issues [40].

### Analysis

Documentary and interview data were uploaded to NVivo™ [43]. We systematically coded each of the interviews and literature review sources according to themes in line with the ACF. These included:

- Stakeholders/actors who influence nutrition policy space
- Interests of the different actors
- Beliefs of the different actors
- Resources, strategies and activities (i.e. how do they use the TIA mechanisms at hand)
- Relationships between actors

For the sake of the analysis, these stakeholders were grouped by ‘type’ in order to develop some theoretical generalisations about shared interests and common sources of power/mechanisms of influence in nutrition policy space through TIAs. We developed an a priori set of actor /stakeholder categories expected to appear in the data. These included: government departments/ministries involved in trade and health, civil society organisations, consumer-citizens, media, trade partners, food and beverage companies and industry lobby groups, trade governance institutions, and standards-setting bodies. We inductively coded based on these categories, continuously updating and modifying the theory deductively through iterative reading of the data. We also coded for *explicit* or *implicit* reference to mechanisms of influence on nutrition policy space and types of power.

Once the data were coded, we then conducted a stakeholder analysis guided by Varvasovsky and Brugha, as described above, organizing data corresponding to each stakeholder-type according to the following characteristics:

- Involvement in the issue (food environment regulation and policy space with respect to TIAs);
- Interest in food environment regulation and policy space with respect to TIAs;
- Influence / power in food environment regulation policy space with respect to TIAs;
- Position with respect to food environment regulations; and
- Impact of food environment regulations and policy space on the actor/stakeholder.
  Coding was conducted in an iterative manner to ensure consistency in the analysis of all source documents. Once all sources had been coded once, the lead author returned to the beginning to review, add new codes and themes that arose later in the coding process, and to identify and rectify any inconsistencies. The senior author reviewed the final coded data in detail.

## Results

Our findings are organised into the following sub-sections: which stakeholders can influence nutrition policy space through TIAs; what are the important networks and relationships between stakeholder groups; what are the primary coalitions operating in the policy subsystem of food environment regulations; how do these coalitions influence (constrain) policy space through TIA mechanisms; and finally, what are the strategies used to preserve policy space for food environment regulations.

### Stakeholders influencing nutrition policy space through TIAs

**Fig. 2:**
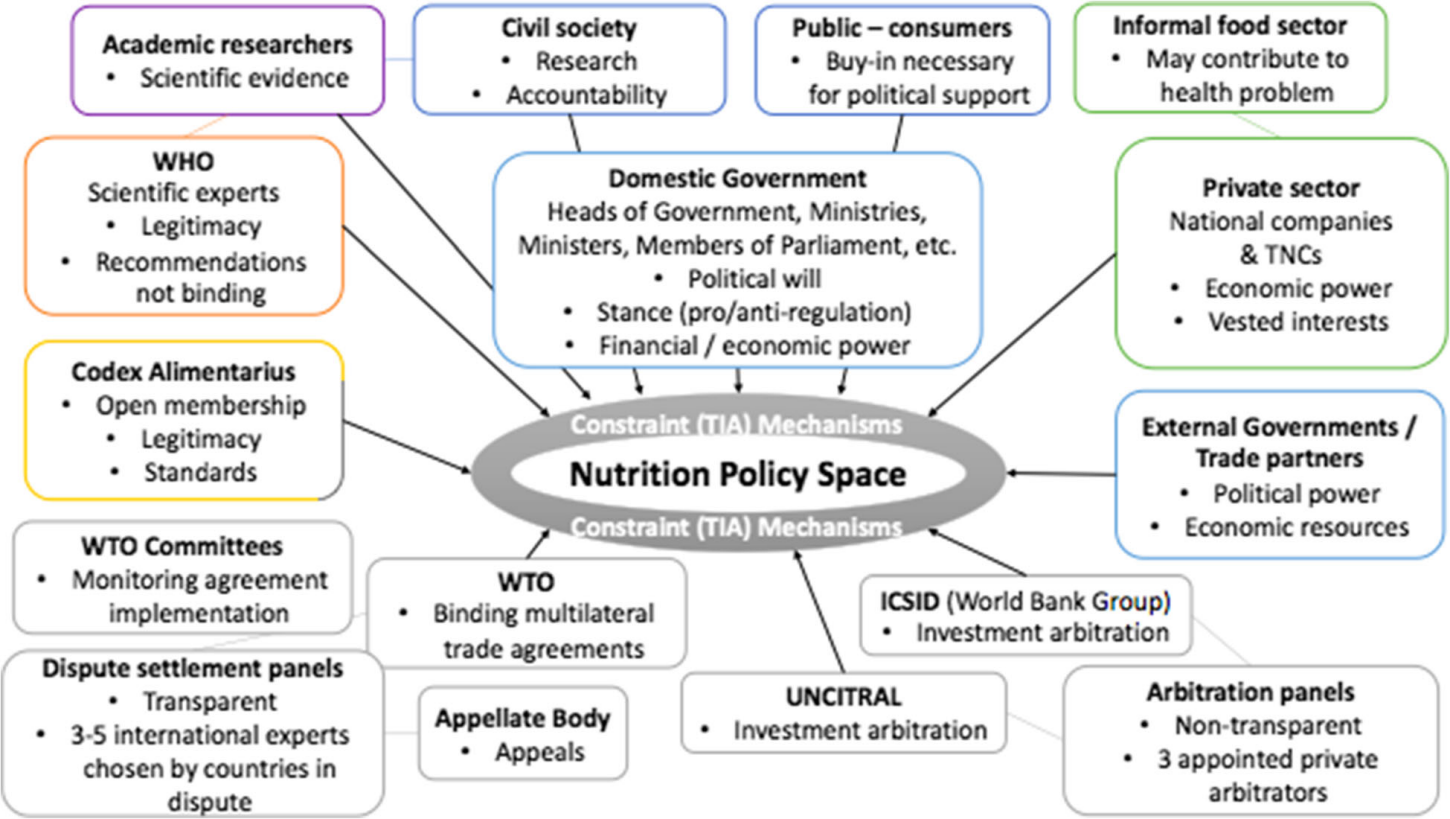
Stakeholder groups within the food environment policy subsystem that potentially influence nutrition policy space. Note: Each box represents a different stakeholder ‘type’ with bullet points highlighting key characteristics that factor into their influence on nutrition policy space. Black arrows represent their ability to influence nutrition policy space. Lines joining stakeholder types represent formal links and areas where there is cross-over between stakeholders. Readers should note that free trade agreements (FTAs) are as important as the WTO agreements and institutional structures, but this diagram only displays the established institutional bodies involved in TIA governance mentioned in the interviews and literature reviewed (and free trade agreements are governed by their Parties, except in the case investor-state dispute settlement (ISDS)

We identified 12 types of stakeholders (understood as single actors or entire institutions) in the literature and interviews that had particularly important roles in the food environment policy subsystem, either supporting policy space for food environment regulation, or contributing to TIA-related constraints (Fig. 2). Influential actors and institutions included: heads of government, politicians, and government Ministries (e.g. of Health, Foreign affairs and trade, Business and industry, etc.); public/consumers, media, and influencers/activists; civil society organisations (CSOs); academics and experts; the World Health Organization (WHO); international standard setting bodies (e.g. the Codex Alimentarius Commission); international trade and investment governing bodies (e.g. the World Trade Organization (WTO), the International Centre for Settlement of Investment Disputes (ICSID) and United Nations Commission on International Trade Law (UNCITRAL)); and the private sector (including TNCs, national companies, and the informal food sector).

### Networks and relationships between stakeholder groups

The actors and institutions within the food environment policy subsystem do not operate in isolation; rather, they form an interconnected network. Formal institutional ties we identified include membership, partnership, and sponsorship. In addition, there were numerous informal ties at play, for instance aid-dependency relationships, lobbying access, or different forms of representation. The formal and informal relationships identified in this analysis that relate to the mechanisms of influence on nutrition policy space have been summarised in Table 2 below.

**Table 2:** Matrix of relationships between stakeholder groups, identified in this study

	Government	CSOs, Media & Academia	Private sector	Trade partners	IGOs
**Government**	Intersectoral engagement between trade and health Balancing political priorities (e.g. export industries, FDI, public health nutrition).	Provides government with information and holds government accountable CSOs represent public (constituency) interests.	Direct lobbying: domestic companies and foreign investors have channels to give input on policy and TIA negotiations. Under BITs and FTA investment chapters foreign investors may be able to raise ISDS disputes.	Bilateral political relationships (e.g. aid) Parties to WTO, BITs, FTAs. May pursue trade challenges to regulation through WTO and FTAs.	WHO and regional bodies provide best practice recommendations for health regulation. Codex Alimentarius provides guidelines for food regulation. WTO and FTAs have governments as parties to (binding) agreements, and governments must implement an adverse dispute outcome by revoking the health measure or face trade sanctions.
**CSOs, Media & Academia**	–	Interaction between different CSO interests e.g. labour unions, health, etc.	Sometimes offers funding (conflict of interest)	–	–
**Private sector**	Regulates business practices within jurisdiction	Monitor business practices	Collaboration in industry associations and lobbying groups	–	–
**Trade partners**	Bilateral political relationships (e.g. aid) Parties to WTO, BITs, FTAs. May pursue (retaliatory) trade challenges to regulation through WTO and FTAs.	May apply pressure through global advocacy.	Direct lobbying: domestic companies and foreign investors and have channels to give input on policy and TIA negotiations. Companies may provide technical expertise and funds to raise trade disputes.	–	WTO and FTAs have governments as parties to (binding) agreements, and governments must implement an adverse dispute outcome by revoking (health) measure or face trade sanctions.
**IGOs**	Membership: WHO (WHA) and regional bodies consist of Member governments. WTO and FTAs have governments as parties to the agreements.	Academia provides formal advice to WHO (technical committees). CSOs can be Observers to Codex.	Industry groups are Codex Observers and advisors to Codex Members.	Membership: WHO (WHA) and regional bodies consist of Member governments. WTO and FTAs have governments as parties to the agreements.	Codex co-sponsored by WHO and FAO. Codex sets standards that are recognised by WTO.

**Abbreviations:** FDI – Foreign direct investment, CSO – civil society organisation, IGO – inter-government organisation, BITs – bilateral investment treaties, FTA – free trade agreement, WTO – World Trade Organization, ISDS – investor-state dispute settlement, TIA – international trade and investment agreements, WHO – World Health Organization, WHA – World Health Assembly, FAO – Food and Agriculture Organization of the United Nations

*Note*: cells describe ways that stakeholders listed on the X axis act upon those in the Y axis

Many of the ties shown in the matrix above represent the first two of Lukes’ dimensions of power: direct/visible influence, and the manner in which institutions are set up. However, the third ‘ideological’ dimension of power, i.e. the underlying core beliefs that pervade the policy subsystem, is also present and contributes to the formation of coalitions we discuss below. We will return to the dimensions of power exerted by the various actors and coalitions in the later section on coalitions’ influence on policy space.

### Coalitions

Recalling that advocacy coalitions are held together by members’ shared beliefs about core policy matters, the data suggested two primary coalitions operating in the policy subsystem of food environment regulations, which we termed “public health nutrition”, and “industry and economic growth”.

### Public health nutrition coalition

We identified a ‘public health nutrition’ coalition operating at national and international levels, typically comprised of Ministries of Health, the WHO and its regional bodies (e.g. the Pan American Health Organization - PAHO), relevant CSOs, public health academic researchers, and championing politicians (e.g. Members of Parliament (MPs), Ministers) and/or celebrity influencers / activists.

Common core policy beliefs were that obesity and diet-related NCDs are a systemic issue (as opposed to a matter of individual choice and responsibility). Actors in this coalition were typically in favour of government intervention in markets for public health purposes, and believed that industry should have limited role and consultation in the regulatory policy process.

Interests that were evident included protecting and promoting the Right to Health (e.g. Ministries of Health, WHO and regional bodies), and enjoying a Right to Health, or Right to Food (e.g. consumer and public interest groups). For example, through: affordability/value for money, and human rights (such as consumer right to information, child health protection, consumer freedom of choice, religious values, etc.).

Issue framing centred around food environment regulations being necessary to reduce systemic drivers of obesity and NCDs, based on WHO recommendations and/or nutrition profiling models, and informed by domestic evidence (e.g. nutrition surveys) or international evidence.

Power/resources: We observed that WHO and regional bodies have legitimacy but low institutional power in the policy subsystem, as recommendations and guidance are weak, non-binding instruments whereas economic treaties are strong and legally-binding. Responses indicated that Ministries of health likewise tend to have less internal government influence than ministries of economy or trade, while CSOs in health and consumer rights tend to be chronically under-funded and have limited power (and institutional mechanisms) to influence domestic and global governance. Championing politicians or celebrity activists (acting as ‘policy entrepreneurs’) were noted to provide an important boost of influence.

### Industry and economic growth coalition

This coalition typically includes trade partner governments (represented in trade forums by Ministries of Commerce/Business/Industry/Foreign Affairs and Trade), private sector organisations (e.g. TNCs, certain national companies, and industry associations), and domestic (internal) government Ministries of Commerce/Business/Industry/Foreign Affairs and Trade.

Common core policy beliefs were that obesity and diet-related NCDs are individual behavioural problems and the responsibility of the consumer; belief in a neoliberal economic model/minimising barriers to commerce and government intervention in markets; scepticism about the need for/appropriateness of food environment regulations; and belief that industry stakeholders should have full consultation and influence in the regulatory policy process.

Interests associated with this group included economic and export growth, lowering costs and increasing (private sector) profit. For example, we noted interests in: livelihoods (e.g. informal sector), unrestricted market access, equal or favourable conditions of competition (e.g. TNCs), growing the economy (e.g. Ministries of Trade, Commerce, also the general public), and promoting the food production and agricultural sector including key exports (e.g. Ministries of Food & Agriculture). Other interests included representing Members or constituents and gaining or maintaining political power (e.g. Head of government, politicians), and reputation/public image (e.g. politicians, champions, companies may want to appear ‘good citizens’ in trade, i.e. rule-compliant).

In terms of issue framing, these actors claimed that food environment regulations: unfairly restrict market access and create inefficiency in global markets, discriminate against their products, are misleading to consumers, deviate from Codex standards (including falsely implying that any precise amount of nutrients is good or bad for health), undermine harmonisation efforts for trade in goods, have insufficient scientific evidence to justify, are more trade restrictive than necessary, and/or go against investors’ ‘legitimate expectations’ of the regulatory environment. An argument coming particularly from coalition actors in the United States was that proposed nutrition regulations *misleadingly* target ‘high in’ products that can be *part of a balanced diet* when consumed in moderation, suggesting public education and physical activity as better solutions to the health problem [44–47].

Power/resources: We observed that TNCs have financial and human resources to engage in the policy process and in pursuing investment claims, and can support governments to represent their interests in trade forums. Private sector stakeholders had multiple institutional avenues to influence nutrition policy space through TIA mechanisms, for example being invited as part of delegations to the Codex Alimentarius or given seats at the table for TIA negotiations. TNCs also had the benefit of operating across borders, often allowing them to pressure multiple governments and wield different TIAs based on which conditions were most favourable, in what is known as forum-shifting. Within governments, Ministries and departments in economic sectors were also perceived to have a high level of internal institutional influence and importance. This economic liberalisation coalition appeared to be dominant, based on its comparative power/resources, and ability to leverage formal and informal relationships. We elaborate five key coalition strategies used to influence nutrition policy space through TIAs below.

### Coalitions’ influence on policy space through TIA mechanisms

This analysis of interview and documentary data indicated five strategies through which private sector stakeholders engage with other industry/economic growth coalition actors to influence nutrition policy space through TIAs: 1) influencing government trade ministries’ internal vetting of regulatory proposals, 2) convincing (and supporting) host governments to raise specific trade concerns and trade disputes, 3) influencing trade agreement negotiations, 4) participation in the Codex standards-setting process, and 5) using transparency and consultation rules to influence nutrition policy making processes.

### Internal vetting of regulatory proposals

Studies have shown that private sector groups have strong ties and access to Ministries of Economy and Trade, who are thus likely to advocate on their behalf in the internal government vetting of regulatory proposals [48, 49]. The types of power evident in this strategy are both institutional and ideological: exerting influence behind-the-scenes through established institutional mechanisms of input, but also influencing the dominant policy beliefs about how things ‘should be’ in an ideological sense. In the policy area of restricting brand advertising to children, a participant expert who had been involved in nutrition policy design explained how industry stakeholders would have direct access to the Ministry of Economy and Foreign Affairs, who would then internally challenge aspects of the regulation these stakeholders disagreed with:

*there are arguments to fight this, but the one you will fight inside the country with the Ministry of Economy (and with the Ministry of Foreign Affairs, who are going to be the go-to for all the problems from international companies), will be that it’s impossible. That the brand is property, and that you are not able to do that.* (P12 – referring to Marketing restrictions)

In the policy area of nutrition labelling, Thow et al. (2019) observed that industry actors have direct access to economic policy makers, who are more influential than health actors in government agenda-setting and decision making [50]. Whereas TNCs had direct access to the policymakers who vet regulatory proposals, there was no equally-powerful public health interest group identified providing input to balance these corporate vested interests. Furthermore, based on the well-known ‘revolving door’ bridging careers in TNCs with those in government and regulatory agencies, we inferred this to be an additional source of internal influence – again, both institutionally and ideologically [51–55].

Our analysis found that this internal regulatory vetting process is now woven into more recent agreements, further entrenching this mechanism of corporate influence. As one prominent legal academic working in this area explained, the Regulatory Coherence chapter in the Comprehensive and Progressive Agreement on Trans Pacific Partnership (CPTPP) applies a Regulatory Impact Assessment (RIA) process, which includes an exposure draft of regulation seeking submissions, and working on a *presumption* of taking the *least intrusive* regulatory approach.(P14 – in reference to Marketing restrictions) Likewise, the Transparency chapter to that agreement requires prior disclosure and consultation with affected industry, in a parallel process where lobbying will occur that may influence the RIA process.(P14) In this example, institutional and ideological mechanisms of influence can be considered as enabling *direct* decision-making power. The more recently negotiated United States Mexico Canada Agreement (USMCA) has Regulatory Coherence provisions that go even further in cementing the RIA and light-handed approach to regulation, and inclusion of industry voice in policy processes [56].

### Raising trade concerns

Governments that are parties to multilateral, regional and bilateral agreements are the complainants in trade disputes brought under the dispute mechanism of those agreements and before their committees, like the WTO TBT Committee. Here, complainants will weigh up the implications of their trade partners’ health measures for their national economy and their major industry stakeholders’ interests. There is evidence of wealthier nations using trade forums as a space to exert and translate political-economic power asymmetries into policy leverage. For instance, while high-income countries have most frequently raised *and* defended WTO challenges related to food, beverage, or tobacco policy, the vast majority of challenges made against LMIC Members have been raised by high-income Members (77.4%) [32]. Several interview participants identified the United States, through the Office of the United States Trade Representative (USTR), as a country with great political sway and power in the international trade regime, whether this is wielded through a formal dispute or other means ‘behind the scenes’ i.e. instrumentally through institutional mechanisms, or appealing to dominant neoliberal ideology.(P8 – Labelling, P14 & P17 – Marketing restrictions). The EU is another powerful trading partner, having raised several of the Specific Trade concerns against front-of-pack nutrition labelling proposals in the WTO TBT Committee [30, 31].

In an analysis of WTO TBT Committee meeting and dispute documents, Barlow et al. (2018) found that country representatives in these forums do occasionally make explicit that their concerns or challenges raised represented the interests of their food and beverage industry, [32] demonstrating how the ‘invisible’ influence of major companies with economic power and political sway is later reflected in the direct decision-making power wielded by governments. They described a Specific Trade Concern discussion in 2006 where a representative from Canada expressed concerns with Thailand’s proposed snack food (health warning) labelling regulations “by noting that ‘the Canadian industry had questioned the scientific merit of the proposed regulation and argued that it discriminated against snack foods’ in a letter to the Canadian government.”( [32] p13) They related a similar TBT Committee discussion in 2013, in which a representative from the United States raised concerns about Peru’s attempts to introduce health warnings on select food and beverage products, stating for the record “that ‘the US pre-packaged food industry has expressed concern over the economic impact of the inclusion of warning statements on a mandatory basis.’”( [32] p13).

TNCs are the complainants in ISDS investment disputes, and often have vast financial resources, technical capacity and personnel to dedicate to fighting a regulation, exerting direct, visible influence on policy space. With such resources, investors may “exploit the loopholes in trade agreements and the structure of the regional economy to gain unjustified advantages and political power,” taking advantage of the uncertainty in interpretation associated with many investment treaty provisions.( [57] p77) One participant had observed that TNCs’ power to exert this type of influence also comes down to *“how much the local economy depends on exports and imports. For countries that are more domestically based, there are much more opportunities [for food environment regulation] than for those who are really open to the market.”*(P11 – referring to Marketing restrictions).

### Negotiation of agreements

In addition to lobbying governments to act on their behalf in trade discussion forums such as the TBT Committee, the private sector is known to lobby its host governments for more favourable conditions when negotiating regional and bilateral trade agreements (through established institutional avenues of input), using strategic framing (i.e. appealing to ideological narratives) [58]. In an analysis of submissions made to the Trans-Pacific Partnership Agreement (TPP) negotiation process, Friel et al. (2016) reported that most of these sought better conditions for market access, harmonisation, and investment protections, for example arguing that the TPP “must not exclude any food product areas and call [ing] for an across-the-board elimination of tariffs and quotas.” ([59] p524) Industry lobbying in this regard frequently appeals to ideals of ‘fairness’, and ensuring equal conditions of competition. Such submissions came from groups like the Canadian Sugar Institute, PepsiCo, Walmart, the National Confectioners Association, Sugar Australia, and the American Sugar Alliance; however, most of the lobbying for easier market access for processed foods was spearheaded by large grocery manufacturer, retail, and food service chain sectors (especially those in the United States) [59]. These major industry bodies had clear capacity and resources to present a strong lobby to their host governments, and in the US for example, had formal avenues of influence through the USTR Trade Advisory Committees [60].

### Governance of international standards

Participation in the Codex Alimentarius standard-setting process was another important identified aspect of industry and economic growth coalitions’ power and influence on TIA-related policy space for food environment regulation, as Codex is the main recognized standard-setting body for food and beverages in trade forums such as the WTO TBT and SPS Agreements. Though country delegations are the actual decision-makers, the private sector has formal and informal institutional avenues for influence within this international standards-setting body. In addition to strong industry lobbying at the national level to influence decisions relating to nutrition policy, food and beverage industry associations may have Observer status to Codex discussions, and private sector representatives are regularly invited to join Member country delegations as technical advisors [50]. A process is currently underway within Codex to develop further guidance on front-of-pack nutrition labelling (FOPL), with the intention of providing some consistency for approaches globally (not necessarily to establish a specific global front-of-pack labelling system). The World Cancer Research Fund (2019) report on ‘building momentum’ for FOPL outlined concerns around private sector input into this process, noting a “significant imbalance on the working group developing the guidelines, with industry overrepresented and the public health community underrepresented,” particularly in light of the clear tensions between public health priorities and food industry objectives to promote trade and consumption.( [61] p9) Thow et al. (2019) found several reasons why public health coalitions have been historically underrepresented among Codex Observers, including lack of awareness and knowledge of Codex governance structures and avenues for engagement, as well as the financial capacity to engage [50]. Their respondents pointed out the cost of attending multiple in-person meetings spanning long decision-making time frames, making consistent participation in discussions difficult [50].

It is safe to assume that this imbalance in representation will be reflected in the dominating frames (ideology) voiced in Codex discussions. Examples of which, as observed by Thow et al. (2019), include framing FOPL as “a ‘restrictive regulatory measure’ that was being implemented in unnecessarily diverse approaches that had associated risks of limiting trade,” and that “labelling is only one intervention and by itself would not ‘solve obesity.’”( [50] p7) Such engagement within the Codex Alimentarius is an example of using institutional power mechanisms as a means to exert ideological influence in international standards-setting. And despite Codex guidelines being by definition voluntary, because they are referenced and interpreted as international standards in several TIA texts, influence within Codex translates into more direct influence over national nutrition policy space.

### Transparency and consultation

Finally, we observed that private sector actors may take advantage of transparency and consultation requirements within TIAs to influence the policy development process. Both the literature and study participants stressed the importance of *appropriate* consultation. Many participants believed that there is space for appropriate industry consultation in the regulatory development process; the question is, at what stage, and whether they are simply consulted or actually engaged in *developing* policy. The World Cancer Research Fund (2019) reported that most countries implementing FOPL to date have engaged industry at some point during the policy making process, with the dominant view that they can provide valuable input on the economic impact to their business of implementing the regulations, and any important technicalities of the regulatory or voluntary measure such as label design [61]. The extent of this industry involvement, they noted, will vary based on the political climate and context of the implementing country. The benefits of such engagement must be balanced with the potential risks of inviting undue industry influence, based on its vested interests (see *supplementary data table*). As explained by one of the legal experts who participated in our interviews,

*They don’t necessarily have to go with industry recommendations, or give them special rights, but giving industry the ability to respond is important. They should get to weigh in on how it is rolled out (e.g. vis a vis their manufacturing cycles), and this should be taken into account. Consultation should be done across the board, including the public. This would apply to investment chapters – e.g. fair and equitable treatment.* (P2 – referring to Labelling)

The World Cancer Research Fund report recommended that governments “set clear guidelines for the type and scope of industry engagement, ensuring the engagement follows the normal legislative consultation procedures required under national law.”( [61] p24).

### Policy space preservation

Public health coalition actors may also act to preserve policy space, and this analysis identified three such strategies. First, CSOs can apply pressure to government leaders for transparency and accountability in public health nutrition policy development, as well as in TIA negotiation [61, 62]. For example, leaked drafts of (secretly negotiated) TPP texts enabled academics and CSOs to conduct health impact assessments identifying the risks posed to public health policy space, before the Agreement was finalised [15, 19, 21]. Second, coalition actors have collaborated to increase their collective influence. In the UK context, Public Health England supported coalition-building among all CSOs working toward obesity prevention, encouraging them to come up with a short list of policy requests. In this case, the establishment of a unified coalition led to a formal institutional avenue to influence decision-making; this coalition then had direct input to policy makers, with a strong unified agenda.(P9 – referring to Nutrient limits) Third, food environment regulations appeared to be ‘legitimised’ through the production and use of scientific research, when academics and experts (including those within CSOs and IGOs such as WHO) provide technical evidence to support advocacy and policy development. The public health nutrition coalition can therefore capitalise on the ideology around scientific evidence and expertise. In Chile, for instance, the campaign to enact the Law on Food Labelling and Advertising was led by a ‘health expert’ politician, with academics helping to inform policy design.(P11 & P12 – referring to Marketing restrictions).

Though our analysis found that public health nutrition coalition actors have the greatest impact when working together, such coordinated action appeared to be less common (or at least the pathways are not as institutionally entrenched) than it is for the industry and economic growth coalition.

## Discussion

### Summary of key findings

This study has described how stakeholders with economic interests influence the ‘food environment policy subsystem’, which spans global and national policy making, particularly with respect to international trade and investment. It has also highlighted the ways in which public health nutrition-oriented stakeholders can and do exert influence, to the benefit of nutrition policy outcomes. We identified 12 types of actors who influence policy space in the food environment policy subsystem. These actors hold various belief systems regarding the economic policy paradigm (e.g. neoliberalism or rights-based), the nature of obesity and dietary NCDs as health problems (e.g. systemic or individual causality), the role of government (e.g. pro- or anti-regulation of markets), and the role of industry in solving the health problem (e.g. full engagement, no role, or limited role). Certain belief systems are also apparent across institutions, in organisational culture.

Based on their core beliefs, these actors and institutions can be broadly grouped into two primary coalitions: 1) a public health nutrition coalition, which is overall supportive of and actively working to enact comprehensive food environment regulation; and 2) an industry and economic growth coalition, which places a higher priority on deregulation and is overall not supportive of comprehensive food environment regulation. The industry and economic growth coalition appears to be dominant, based on its relative power, resources and coordination. However, the public health nutrition coalition maintains influence through individual activism, collective lobbying and government pressure (e.g. by CSOs), and expert knowledge generation.

Our analysis suggests that the industry and economic growth coalition is highly capable of leveraging networks and institutional structures to its advantage, as well as promoting and entrenching a set of pervasive beliefs within the policy subsystem, and is a formidable source of opposition acting to constrain nutrition policy space, including through TIAs.

There are opportunities for the public health nutrition coalition to strengthen its influence in the support of nutrition policy space.

### Interpretations – comparison with contemporary scholarship

These findings are consistent with emerging literature on the roles, activities and power of stakeholders in policy change within the food environment policy subsystem. In particular, this study follows a growing body of research applying the ACF to nutrition policy change questions in global contexts, noting tensions between public health/nutrition coalitions, industry/economic coalitions, and in some cases food security coalitions [63–67]. As previous studies suggest, at the heart of this tension is a difference in core policy beliefs. Because food systems have been principally designed in economic terms (i.e. to generate profits and to feed those who can pay), the result is a system that is heavily oriented toward profits for individuals and companies within the commercial food system, and economic growth, exports and productivity for countries [68]. The current international trade and investment system has been characterised by: an overriding neoliberal ideology, which dominates over public health values; an institutional structure of ‘new constitutionalism’ (in which state sovereignty is transferred to supranational governance structures), undermining public health legitimacy in trade and investment spaces; and disparity in power and resources between economic actors and public policy actors, constraining the capacity of public health actors to influence TIA negotiations [69].

In terms of solutions, some experts propose targeting formal structures, for instance revising some of the language in TIAs, reforming ISDS processes, and establishing limits on private sector participation in TIA negotiations to avoid conflicts of interest [27]. Other proposed solutions involve more coordinated action on behalf of nutrition policy supporters. Examples include the establishment of academic networks – to generate research not just in justification of food environment regulations, but to create monitoring systems for government accountability [70]. Such research is a valuable input for advocacy, and CSOs have been lauded as the ‘sleeping giants’ with potential to tip the scales of influence by translating knowledge into activism [68]. As their resources for these activities are typically scarce, experts have called on philanthropic organisations to fund this type of work. It has also been suggested that coalition building with social movements around climate change and food sovereignty may help to disrupt the power dynamics standing in the way of food systems transformation [68].

Lencucha and Thow (2020) point out that in recent years, some governments and international economic agencies have begun to re-evaluate the status-quo of narrow economic rationality that places economic growth above health, environment or other social goals, providing ‘windows of opportunity’ for transforming how governments approach health-harmful industries such as unhealthy food, alcohol and tobacco [71]. This emerging shift presents the public health community with openings to work with different sectors of government to reimagine policy mandates (encouraging a whole-of-government imperative for sustainable development), and to closely examine the institutional structures and governance processes that stall progress towards improved health outcomes [71].

### Implications for a public health nutrition coalition: how to strengthen nutrition policy advocacy

This analysis suggests potential entry points for the public health nutrition coalition to focus efforts, leverage support and build capacity for preserved/increased policy space.

**Fig. 3:**
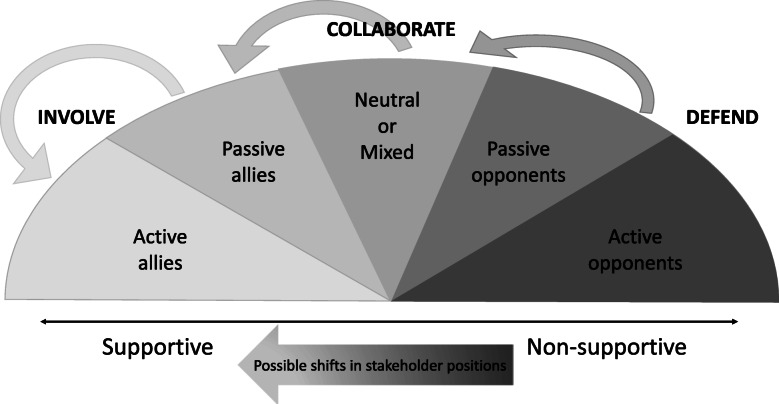
Strategies for managing a spectrum of stakeholders according to their organisational policy positions. Adapted from Varvasovsky & Brugha (2000) [37] and www.powershift.org (n.d.) [38]

Vavrasovsky and Brugha (2000) recommend different strategies (involve, collaborate, defend, or monitor) for ‘optimally’ managing different stakeholders, according to their positions on the issue in question [37]. This also ties into the advocacy Spectrum of Allies theory that separates policy subsystem groups into active allies, passive allies, neutrals, passive opponents, and active opponents. It asserts that campaigns are not won by targeting one’s actively resistant opponents, but rather by shifting the attitudes and perceptions of passive supporters and neutral observers [38]. This implies that the public health nutrition coalition could maximise its impact by considering this spectrum within the categories of stakeholders identified, especially those with neutral or diverse/mixed positions, and focusing advocacy efforts on shifting those in the middle of the spectrum (Fig. 3).

In the simplest terms, this suggests that public health nutrition coalitions should involve more groups who are, or could be, supportive of food environment regulation. Some of these groups may be non-mobilised. As indicated by the data, this could include the mainstream media (as an enabling force), and a wider scope of CSOs i.e. public interest groups with an interest in food systems transformation. Swinburn (2019) suggests, for example, that “for some countries and regions, narratives around food security, food sovereignty or malnutrition may have greater currency, but whatever the narrative, it needs to be inclusive of the many groups and people who care about food in different ways.” ([68] p6) This type of mobilisation is important because visible support is needed to motivate governments to act.

Varvasovsky and Brugha (2000) suggest collaboration with the diverse-position groups [37]. Here, several questions arise. What does collaboration mean, exactly? Is it ‘safe’ for public health nutrition coalitions to collaborate with TNCs that are less resistant to regulation—due to different underlying company philosophies, for example? How could we make it safe to do so? This is a particularly salient question, when a large proportion of current political discourse on food systems frames the food industry as an essential “part of the solution,” and given there are very different implications between being involved in implementing the solutions, and in designing them.

Some critical public health experts argue that food and beverage companies, under the current business-as-usual conditions, can only go as far as their customers and shareholders (i.e. their institutional structures) will allow, and carrying front-of-pack nutrition warning labels, increasing prices to dissuade consumption, or internalising the negative externalities of their unhealthy commodities based on ‘public-spiritedness’ alone is not conceivable [68]. In that vein, “the single most powerful thing food industries can do is to support government policy attempts to create healthier, more sustainable food systems and, especially, *refrain from undermining them*” (emphasis ours).(67 p8) On the other hand, there may be opportunities for a public health nutrition coalition to build bridges with the economic sector in new and constructive ways that explicitly remove support from unhealthy commodity-producing industries in favour of industries, consumer products and modes of production that are sustainable, health-promoting, *and* contribute to the economy [71]. In this regard, we posit that understanding the influential actors and their beliefs can give insights regarding how, and with whom, to engage effectively. There is, in any case, a clear need for establishing terms of engagement outlining appropriate involvement for private sector stakeholders in the regulation of food environments.

We can also consider strategic action in terms of what kind of change is within our realm of influence. For example, the Lancet Commission on Obesity report (2019) explains that policy inertia in obesity prevention is contributed to in large part by power asymmetries in food systems, including corporate-captured or inept government, food industry opposition, and weak civil society [72]. Unfortunately, the average public health actor cannot change the opposition from the food industry. Changing the corporate capture of governments is a long-term endeavour that will require concerted efforts. The public health nutrition coalition may, however, be able to influence outcomes in the shorter term by strengthening the voices of CSOs that can help to hold governments accountable.

### Specific recommendations for public health nutrition actors

A number of strategic actions may be worth pursuing by public health nutrition advocacy coalition actors to preserve nutrition policy space, at both the international (IGO)-level, and domestic levels, which we briefly discuss below.

### International (IGO)-level actions

This analysis suggests three avenues for action at the international level. First, the legitimacy associated with scientific expertise in the TIA interpretation space indicates that the production of robust evidence by public health nutrition researchers and credible IGOs with a public health nutrition mandate (such as WHO and its regional offices) will enable justification of food environment regulations in international trade and investment forums. Generation of this evidence will be enabled by strategic planning in terms of allocating resources to strengthening the evidence base where it is most needed. Second, the uneven influence observed between coalitions in international standard-setting indicates that public health nutrition policy makers and CSOs could work to bring greater/stronger public health presence within Codex, specifically in standard development for *nutrition labelling*. Third, in light of the opportunities for health policy space preservation gained from obtaining access to secret negotiation texts, there may be opportunities for the public health nutrition advocacy coalition to push for transparency in investment arbitration.

### Domestic-level actions

Based on this analysis, we also propose the following avenues for action at the domestic level to preserve nutrition policy space with respect to TIAs:

First, this research demonstrates the importance of positive public opinion. There is an opportunity for public health nutrition researchers and CSOs to foster this through broad public engagement (already being done in many countries), to include more potentially supportive actors. For example, if media is non-mobilised (i.e. uninterested), reporters could be engaged (and trained) to ensure balanced and factual reporting on the public health nutrition implications of TIAs and the trade implications of nutrition regulations. In addition, there may be opportunities to collaborate with trade analysts and activists not yet considering the food systems and nutrition angle.

Second, the imbalance of power between sectors of government indicates that public health nutrition advocacy coalition members within government could build capacity within Ministries of Health to more effectively engage with trade Ministries on questions of TIA negotiation (or RIAs), and to translate to them the public health importance of policy proposals. Researchers, CSOs and policy makers could advocate for publicly-accessible Health Impact Assessments to be carried out as part of any TIA negotiation process, which include an examination of potential constraints on policy space for regulating food environments (among other public health objectives).

Third, this research highlights the potential conflict of interest arising from institutional arrangements that favour private sector input within the policy subsystem. Researchers and CSOs should advocate that their governments commit to setting clear guidelines for the type and scope of industry engagement in domestic policy making processes (including trade and investment agreement negotiations), in accordance with whatever normal legislative consultation procedures are required under national law [61]. By the same token, transparent institutional processes could be established to give public interest organisations and academic experts equal opportunities to input into policy processes. Schram et al. (2019) suggest that governments could use the Framework of Engagement with Non-State Actors, developed by the WHO, as a template to define their own terms of engagement with non-state actors [27]. This could include laying out the risks of engagement, potential conflicts of interest, the types of interactions permissible, and required transparency [27].

Finally, there appear to be a number of opportunities to strengthen civil society. CSOs could identify ‘passive ally’ groups at the domestic level who could be brought on board to the public health nutrition advocacy coalition’s cause of food environment regulation (including, as mentioned above, groups engaged in international trade and investment with respect to other health and social issues). Interested CSOs could build their inter-sectoral capacity to better understand the implications of increased trade and investment liberalisation on nutrition policy space. CSOs working toward nutrition regulations for NCD prevention could coordinate their advocacy agendas, for instance focussing on a few key ‘asks.’ New and/or unconventional sources of funding and resources could be channelled to their activities. Bloomberg Philanthropies’ Food Policy Program, for example, has funded CSOs working toward obesity prevention in Mexico, the Caribbean, Colombia, Brazil, South Africa, and the US [73]. Finally, specific to labelling, training and resources could be sought to enable greater/stronger engagement within Codex, at both the national and global level [74].

### Strengths and limitations

This study presents a comprehensive and systematic analysis of the stakeholders within the policy subsystem of food environment regulation that potentially influence policy space with respect to TIAs, and the connections between them, guided by established theoretical frameworks. This analysis is the first to comprehensively collate this information to answer the specific question of how actors and institutions play a role in the constraint or preservation of government policy space for food environment regulation, through TIAs.

Due to the nature of the data collected (i.e. perspectives from global literature and international experts), we have had to make broad generalisations for groups of stakeholders, though one could expect some heterogeneity within stakeholder categories, and for whole groups in different contexts. Finally, it is analytically challenging to determine the ‘true’ interests of actors and to disentangle ties of influence as this is inherently a matter of perception, and often these are concealed. As such, the analysis presented in this article serves best as a guide to help make sense of the agency of various stakeholders within the global nutrition policy sphere with respect to TIAs. A more in-depth analysis would need to be applied in order to answer specific policy space questions in specific contexts.

## Conclusions

Our analysis identified two primary competing advocacy coalitions engaged in either constricting or supporting policy space for food environment regulation globally. Our findings suggest that the economic growth coalition is dominant, highly capable of leveraging networks, institutional structures and dominant ideologies to its advantage, and is a formidable source of opposition acting to constrain nutrition policy space, including through TIAs. Opportunities for the public health nutrition coalition to strengthen its influence in the support of nutrition policy space include strategic evidence generation, and coalition-building through broader engagement and capacity-building.

## Supplementary Information

**Additional file 1: Table S1.** Characteristics of stakeholder types relevant to the food environment policy subsystem with respect to international trade and investment agreements.

## Data Availability

All data generated or analysed during this study are included in this published article in and in its supplementary information files.
