# Supplementary material for: Who influences nutrition policy space using international trade and investment agreements? A global stakeholder analysis
Source: Global Health. 2021 Oct 2;17:118. doi: 10.1186/s12992-021-00764-7 (PMC8487514; doi:10.1186/s12992-021-00764-7)
Supplement: Supplementary file 1 — Additional file 1: Table S1. Characteristics of stakeholder types relevant to the food environment policy subsystem with respect to international trade and investment agreements. [file 12992_2021_764_MOESM1_ESM.docx]

Table 1-S. Characteristics of stakeholder types relevant to the food environment policy subsystem with respect to international trade and investment agreements.

| Stakeholders | Characteristics | | | | |
| --- | --- | --- | --- | --- | --- |
|  | Involvement in the issue: nutrition policy space for food environment regulation (with respect to TIAs) | Interest in the (food environment regulation) nutrition policy (High / Medium / Low / Mixed) | Influence / power in nutrition policy space  (High / Medium / Low / Mixed) | Position regarding food environment regulation (Supportive / Mixed / Non-mobilised / Opposed / Marginal) | Impact of the food environment regulation on stakeholder |
| Head of Government regime | Public figurehead of regulation.  Government in power would need to defend regulation in any trade/investment disputes and/or sanctions.  May also have ties to food and beverage TNCs within country. For example, food and beverage industry groups often donate to political campaigns. | Medium: one of many political issues to be balanced.  Ideological – party values. Constituency-based - maintenance of power. | High (within country)  Power with respect to other international stakeholders may depend on country resources: economic, political, and capacity (e.g. to assess legal basis or implications of any threats made). | Mixed; however, this study operates under the assumption that the governing power do in fact want to implement this type of nutrition policy. | Alignment with personal/party core beliefs.  Reputational risk.  Potential cost of ISDS and awards.  May impact their re-election potential. |
| Politicians (such as Members of Parliament – MPs, and Ministers) | Will vote for or against a regulatory Bill.  May also propose and push for Bills etc. | Diverse / Mixed.  Some personal interest /ideological; some constituency-based (implications for re-election). | Medium / high. | Diverse / Mixed.  *May depend upon whether/how well they understand the nutrition policy issue.* (P19 - Nutrient limits) | Personal – alignment with core beliefs, and personal/family/ community health impact.  Political – to gain or lose favour among constituents. |
| Ministries of Health | Recommend and develop technical regulations to improve the healthiness of the food environment. | High.  Part of mandate to protect and promote population health | Medium.  May be relatively lower among government ministries (depending on regime) | Supportive. Pro- regulation. | Alignment with institutional mandate to protect and promote population health. |
| Ministries of Commerce / Business / Industry / Foreign Affairs and Trade | May raise concerns about the impacts of food environment regulations on international trade and domestic economy.  Represents the interest of private industry (domestic and/or foreign trade partners and investors). | Medium-Low: one of many policy issues relevant to economic growth. | High.  May be relatively high among government ministries (depending on regime). | Potentially opposed to food environment regulation. | RIAs may highlight negative impacts on trade or foreign investment.  Reputational concerns for being seen as rule-compliant, in order to be able to challenge other trade partners from a position of strength. Potential concern about setting precedents that impact exports or investment offshore. |
| Ministries of  Food and Agriculture | Potentially affected by standards for fat content in meats, and regulations involving dairy products. | Generally low interest, unless it relates to a key domestic industry (e.g. meat, dairy, palm oil (saturated fats), sugar, corn) – then high. | High.  Typically have strong industry lobby. | Marginal or Opposed. | Potential economic impact if regulations target important domestic industry products. |
| Trade partner governments | May initiate discussion of concerns with regulation (bilaterally or in wider forums e.g. TBT Committee), or formal dispute processes, on behalf of domestic industry bodies. | Medium-High.  Interest in protecting their domestic industries & companies. Likely to promote harmonisation to match their regulations. | Diverse / Mixed.  Depends upon country’s political and economic power and resources (including legal capacity). | Diverse / Mixed. | Impact to domestic industries and the national economy.  Concern about precedents that might be set or reinforcing a trend to certain policies that would adversely affect exports and investors offshore. |
| Private sector | The subject of food environment regulations.  May be private or publicly traded companies. | High.  Profits. Returns to shareholders. Market access. Conditions of competition.  May need to change production, communication and sales practices (reformulation, labelling, marketing and advertising) to comply. | Medium-High.  Depends upon size and resources of company/industry and, in the case of lobbying governments, contribution to national economy.  Higher influence when organized into industry associations. | Generally opposed, though not homogeneously.  Some may be acceptant (non-mobilised). | Decreased profits, returns to shareholders. |
| *Anti-regulation industry lobby groups* | *Companies subject to regulations* | *High interest.*  *Values: Change-resistant* | *Negative influence*  *Power will differ* | *Opposed* | *Prompts (unwelcome) changes in company practices* |
| *Pro-regulation companies* | *Subject to regulations. Involvement in nutrition policy issues likely to be on case-by-case basis* | *High interest.*  *Values: Company philosophy of being progressive, ahead of trends* | *Positive influence*  *Power will differ* | *Acceptant, embracing* | *Prompts changes in company practices, may gain competitive advantage* |
| Informal sector | Can be large source of sodium /fats /sugars in diets, especially in LMICs, rural areas, or lower-income areas of HICs.  Their buy-in and compliance is important for achieving health objectives, but they are difficult to regulate (especially in terms of enforcement).(P9 – Labelling, P21 – Nutrient limits) | High.  Regulation directly affects their livelihood. | Low.  Small sector with relatively little impact at stake at national scale. | Mixed? Potentially opposed to food environment regulation. Likely non-mobilised.  Might be convinced of the greater benefit.(P9 – Labelling) | Regulations, if followed, may have an impact on sales.  Though, potential to experience nutrition /health benefits to self, family, community (long term). |
| Public-consumers | Consumers on the receiving end of technical regulations seeking to improve food environments. | Diverse / Mixed.  Will depend upon personal beliefs, e.g. Value for money. Human rights (e.g. consumer right to information, child health protection).  Consumer freedom of choice. Religious values. etc. | Individual: Low  Collectively: Medium-High.  Collective public opinion can increase or decrease political will to impose regulations (though very little individual power or influence)  Influence of special interest groups depends on size of community, relative to constituency. | Diverse / Mixed (Supportive, Non-mobilised, Opposed). | Stand to end up with reduced access, affordability and/or marketing of unhealthy food and beverages.  Potential improvement in health outcomes (long term) |
| Media | Disseminates information, influences public opinion. | Low-Medium.  Primary interest in maintaining readership: to be ahead of the news.  Potential vested interest in retaining advertising revenues (in the case of advertising restrictions).  Depending on media ownership, may have other vested interests. | High.  Can have significant influence over public opinion. | Likely marginal, but may not be ‘neutral.’ | Potential impact on advertising revenues (in the case of advertising restrictions) |
| Celebrity influencers / lobbyists | High-profile, vocal supporter; influencing public education and opinion (also industry, informal sector and government). | High.  Personal values.  Professional and financial gain or risk (fame, reputation, sponsorship). | Medium.  Can have significant influence over public opinion. | Mixed.  While there may be strong advocates for nutrition policies, the role of influencers in marketing on behalf of TNCs is significant. | Alignment with personal values;  Potential professional gain or risk. |
| Civil society organisations  (e.g. public-interest NGOs, charities) with a health, food and nutrition, consumer rights, or trade justice focus | Provide research and evidence; lobbying and advocacy;  hold government accountable. | High.  But tend to all want different things; coordination of agendas is key.(P19 - Nutrient limits) | Medium-High.  Could potentially be high, but will depend on resources. Generally, CSOs are weak and poorly-resourced. However, consumer organisations may be well-resourced and networked internationally. | Some supportive.  Many likely remain non-mobilised. | Alignment with institutional mandates (e.g. to promote child health, human rights / consumer rights, food security, food sovereignty). |
| Academics and (public health nutrition, public health law) experts | Provide scientific evidence and expert input to:  Governments (to guide policy making), and  CSOs (to support advocacy)  Research funding often comes from government agencies, IGOs, or philanthropic organisations. Funding is also often offered by private sector – potential conflict of interest. | High.  Personal and professional interest. | Medium.  Limited research funding. Capacity to influence policy space depends on who is listening. | Supportive. | Alignment with personal values. Potential professional gain or risk; e.g. refusal to accept industry money (to avoid conflict of interest) may mean less research funding. |
| World Health Organization (WHO) | IGO with mandate to protect and promote global health. Comprised of 194 Member States who appoint delegates to the World Health Assembly, the WHO’s supreme decision-making body.  Operations carried out by technical experts, who provide recommendations and guidelines.  Regional and country offices are often quite engaged at country level in supporting nutrition policy, particularly PAHO with respect to the challenges that have occurred in Latin America. | High.  Values: Health promotion. Objective scientific evidence.  Represent Member countries’ interests. | Medium-High.  Well-respected and recognized as the prime international voice for health. However, recommendations are not binding. | Supportive. Pro-regulation.  In its mandate to represent *all* member countries’ interests, WHO may have a harder time officially promoting more progressive guidelines due to pushback from some member countries (on behalf of domestic industry lobbies). | Alignment with institutional mandate. |
| Codex Alimentarius Commission (CAC) | Established jointly by the WHO and FAO to protect consumer health and promote fair practices in food trade. 189 Codex Members (188 countries, plus the European Union), and 236 Observers (57 IGOs, 163 NGOs, 16 UN) who may attend meetings and put forward their views at every stage of the standard-setting process.(217)  The Codex is a collection of standards, guidelines and codes of practice adopted by the CAC. | High.  Codex is explicitly involved in food labelling and food safety.  Guidance given on compositional requirements of food to be nutritionally safe, general labelling of foods, and health or nutrient claims. | High.  Codex standards are officially recognised by WTO agreements (e.g. WTO TBT and SPS). | Diverse / Mixed.  Diverse membership means mixed positions regarding regulation. Hence, processes to establish new standards (e.g. on FOPL) are very slow. | None (N/A)  Business-as-usual |
| WTO Committees, e.g. TBT Committee | Monitor the implementation of WTO agreements. There are various (e.g. on TRIPS and Services), but the TBT Committee is historically the most active.  The TBT Committee, for instance, is made up of TBT Member country representatives who discuss policy notifications to raise any STCs regarding potential impacts of proposed regulations. | Medium-High.  Members have own national economic interests at heart. Their interest will depend upon impact to domestic industry. | Diverse / Mixed.  Economic power of Member States (e.g. HIC vs LMIC) is associated with who raises concerns, and may influence how Members respond.(62) | Diverse / Mixed.  Position of Members with respect to specific nutrition policy proposals may depend upon bilateral political relationships (e.g. alliances, political pressure). | None (N/A)  Business-as-usual |
| WTO - Dispute settlement panels | Panels consist of 3-5 legal or trade experts, often chosen by the countries in dispute.  Dispute settlement procedure is relatively transparent. | Low.  Supposed to be objective. | High.  Reports are effectively legally binding unless appealed, as they must be rejected by all Members | ‘Neutral’ / Non-mobilised. | None (N/A)  Business-as-usual |
| WTO Appellate Body | Appeals from dispute settlement panel decisions.  Procedures are relatively transparent. | Low.  Supposed to be objective. | High.  Final say in cases that are appealed. Reports are effectively legally binding, as they must be rejected by all Members. | ‘Neutral’ / Non-mobilised. | None (N/A)  Business-as-usual |
| Investment Arbitration panels | Investment dispute arbitration panels are appointed ad-hoc, and comprised of three private sector arbitrators, 1 appointed by the investor and 1 by the state and they appoint the chair.  Most arbitration procedures are not transparent | Low.  Supposed to be objective. However, arbitrators have an interest in being seen as pro-investor to maintain business. | High.  Awards of financial compensation are legally binding. | ‘Neutral’ / Non-mobilised. | Professional reputations at stake: to keep business, arbitrators are often seen as pro-investor. However, in light of global debates and scepticism around IIAs and policy space, arbitration panels may be more reluctant to make decisions against public health nutrition policies and other controversial issues. (P13 - Marketing restrictions) |
